# Gastric Corpus Mucosal Hyperplasia and Neuroendocrine Cell Hyperplasia, but not Spasmolytic Polypeptide-Expressing Metaplasia, Is Prevented by a Gastrin Receptor Antagonist in H^+^/K^+^ATPase Beta Subunit Knockout Mice

**DOI:** 10.3390/ijms21030927

**Published:** 2020-01-31

**Authors:** Kristin Matre Aasarød, Helge Lyder Waldum, Astrid Kamilla Stunes, Arne Kristian Sandvik, Arnar Flatberg, Patricia Mjønes, Unni Syversen, Ingunn Bakke, Reidar Fossmark

**Affiliations:** 1 Department of Clinical and Molecular Medicine, Faculty of Medicine and Health Sciences, NTNU—Norwegian University of Science and Technology, 7491 Trondheim, Norway; kristin.aasarod@ntnu.no (K.M.A.); helge.waldum@ntnu.no (H.L.W.); kamilla.stunes@ntnu.no (A.K.S.); arne.sandvik@ntnu.no (A.K.S.); arnar.flatberg@ntnu.no (A.F.); patricia.mjones@ntnu.no (P.M.); unni.syversen@ntnu.no (U.S.); ingunn.bakke@ntnu.no (I.B.); 2 Department of Gastroenterology and Hepatology, Clinic of Medicine, St Olav’s University Hospital, 7491 Trondheim, Norway; 3 Centre of Molecular Inflammation Research, Faculty of Medicine and Health Sciences, NTNU—Norwegian University of Science and Technology, 7491 Trondheim, Norway; 4 Department of Pathology, Clinic of Laboratory Medicine, St Olav´s University Hospital, 7491 Trondheim, Norway; 5 Department of Endocrinology, Clinic of medicine, St. Olav´s University Hospital, 7491 Trondheim, Norway; 6 Clinic of Medicine, St. Olav’s University Hospital, 7491 Trondheim, Norway

**Keywords:** gastrin, netazepide, SPEM, acid inhibition, neuroendocrine cells

## Abstract

Proton pump inhibitor use is associated with an increased risk of gastric cancer, which may be mediated by hypergastrinemia. Spasmolytic polypeptide-expression metaplasia (SPEM) has been proposed as a precursor of gastric cancer. We have examined the effects of the gastrin receptor antagonist netazepide (NTZ) or vehicle on the gastric corpus mucosa of H^+^/K^+^ATPase beta subunit knockout (KO) and wild-type (WT) mice. The gastric corpus was evaluated by histopathology, immunohistochemistry (IHC), in situ hybridization (ISH) and whole-genome gene expression analysis, focusing on markers of SPEM and neuroendocrine (NE) cells. KO mice had pronounced hypertrophy, intra- and submucosal cysts and extensive expression of SPEM and NE cell markers in the gastric corpus, but not in the antrum. Numerous SPEM-related genes were upregulated in KO mice compared to WT mice. NTZ reduced hypertrophia, cysts, inflammation and NE hyperplasia. However, NTZ neither affected expression of SPEM markers nor of SPEM-related genes. In conclusion, NTZ prevented mucosal hypertrophy, cyst formation and NE cell hyperplasia but did not affect SPEM. The presence of SPEM seems unrelated to the changes caused by hypergastrinemia in this animal model.

## 1. Introduction

Proton pump inhibitors (PPIs) are widely used in the management of acid-related gastrointestinal diseases such as peptic ulcers and gastro-esophageal reflux. The increase in PPI use has been well described in many countries in recent decades [1,2]. The consequences of long-term acid inhibition, however, are not fully known. In humans, the first accepted gastric side effects of PPI use was development of fundic gland polyps [3,4]. Based on animal studies, an increased risk of gastric neuroendocrine (NE) tumors developing from enterochromaffin-like (ECL) cells and carcinomas has been predicted since the 1980s, and more recently several epidemiological studies have found that patients using PPIs have an increased risk of gastric cancer [5,6,7]. Numerous studies suggest that hypergastrinemia, which is a common factor found in many conditions with increased cancer risk, is a key element in gastric carcinogenesis of the corpus and fundus [8,9]. In response to gastric luminal contents and hypoacidity, gastrin is released from antral G cells and stimulates function and proliferation of ECL cells where the gastrin receptor is located [10]. Gastrin release is also regulated by gastrin-releasing peptide (GRP) and GRP receptor (GRP-R) expression in the gastrointestinal tract has been located to the antrum [11]. The GRP-R is also expressed in a range of tissues outside the gastrointestinal tract [12] as well as in various types of cancer [13,14].

One proposed precursor lesion to gastric cancer, in addition to the more established intestinal metaplasia (IM), is spasmolytic polypeptide-expression metaplasia (SPEM). It has been postulated that chief cells may dedifferentiate or transdifferentiate into SPEM [15], which is characterized by the expression of glands with mucous-containing trefoil factor 2 (TFF2), also named spasmolytic polypeptide. MUC6 and clusterin are markers of SPEM and numerous other genes are differentially expressed in tissue containing SPEM. SPEM has been studied extensively in mice given an agent causing parietal cell atrophy (DMP-777, L-635 or tamoxifen) [16]. The role of gastrin in SPEM development is not completely understood. In gastrin knockout (KO) mice given DMP-777, development of SPEM is accelerated [17], but remarkably, gastrin receptor KO mice develop SPEM after DMP-777 administration following a similar timeline compared to wild-type (WT) controls [18]. The role of histamine has also been investigated and a proportion of histidine decarboxylase (HDC) KO mice develop SPEM spontaneously and have accelerated development of SPEM after DMP-777 administration [19]. Histamine 2 receptor KO mice given DMP-777 develop SPEM similar to WT controls [18].

The H^+^/K^+^ATPase beta subunit KO mouse is a model of long-term PPI use, gastric hypoacidity and secondary hypergastrinemia [20,21,22]. KO mice develop marked hyperplasia of the oxyntic mucosa with intramucosal cystic changes and ECL cell hyperplasia, but only a few develop gastric carcinoma [21].

In the present study, we have examined the effects of the gastrin receptor antagonist netazepide (NTZ) in KO and WT mice, with particular focus on NE hyperplasia and SPEM markers.

## 2. Results

During the study period, two animals died—one in the WT/NTZ group and one in the KO/PEG group—leaving 11 mice in the WT/PEG and KO/NTZ groups and nine in the two other groups. No differences were observed in animal welfare between the different groups. 

### 2.1. KO Mice Had Elevated Intragastric pH and Plasma Gastrin

**Figure 1:**
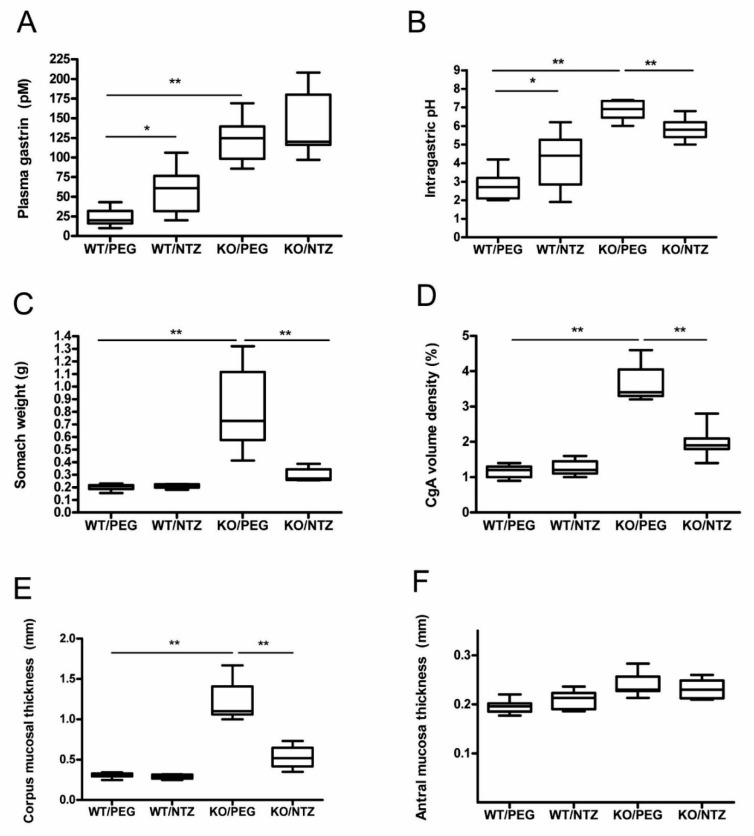
Intragastric pH (**A**), plasma gastrin (**B**), stomach weight at termination (**C**), chromogranin A (CgA) volume density (**D**), corpus (**E**) and antral (**F**) mucosal thickness in H^+^/K^+^ ATPase beta subunit knockout (KO) mice and wild-type (WT) controls given netazepide (NTZ) or polyethylene glycol (PEG) as vehicle.

The mice in both KO groups had significantly higher intragastric pH than WT/PEG (*P* = 0.001) and WT/NTZ (*P* = 0.003) mice. WT/NTZ mice had higher intragastric pH than WT/PEG (*P* = 0.044). Both KO groups had high median gastric pH, but lower pH levels were observed in the group receiving NTZ compared to vehicle (*P* = 0.001) (Figure 1A). Gastrin levels were higher in the KO groups compared to the WT groups (*P <* 0.001), and the levels were also higher in WT/NTZ mice compared to WT/PEG (*P* = 0.005). No significant differences between the two KO groups were observed (Figure 1B).

### 2.2. NTZ Reduced Stomach Weight and Oxyntic Mucosal Thickness in KO Mice

Stomach weight was higher in the KO/PEG group than in all other groups (*p* = 0.001), as expected from the known effects of hypergastrinemia. KO/NTZ mice had lower stomach weight than KO/PEG mice, reflecting the effects of gastrin receptor antagonism, but still higher than both WT groups (*p* = 0.001) (Figure 1C). Oxyntic mucosal thickness was lower in KO/NTZ mice compared to KO/PEG, whereas there was no difference between the WT/NTZ and WT/PEG groups (Figure 1D). Antral mucosal thickness did not differ between the groups (Figure 1F).

### 2.3. NTZ Reduced Intramucosal Cysts and Invasions below the Muscularis Mucosae in KO Mice

**Figure 2:**
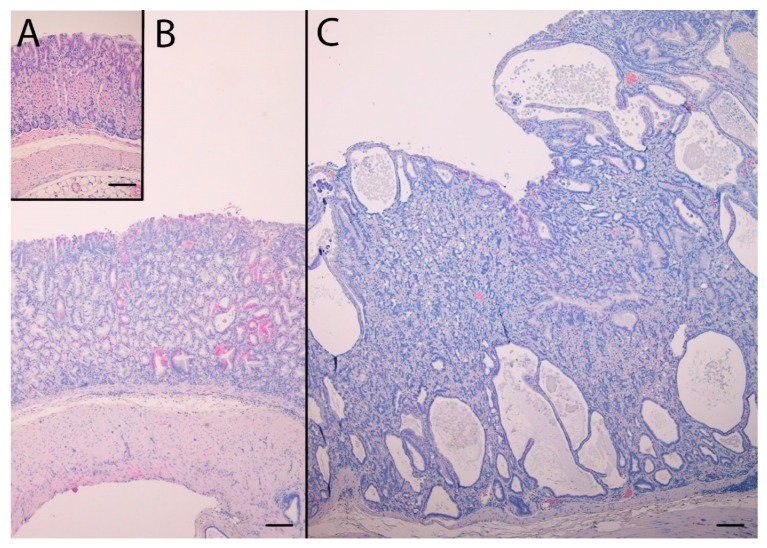
Hematoxylin and eosin stained sections of the oxyntic mucosae from WT/PEG mice (**A**), KO/NTZ mice (**B**) and KO/PEG mice (**C**). There is marked hyperplasia and intramucosal cysts in KO mice that are reduced by NTZ. Scare Bar = 100 µm.

In addition to pronounced hyperplasia of the oxyntic mucosa, KO mice had intramucosal cysts and half of the animals had invasions of the muscularis mucosae with benign appearance, often in the proximity of vascular structures also penetrating the muscularis mucosae. The histopathological changes are presented in Table 1. NTZ reduced the number of intramucosal cysts as well as submucosal invasions in KO mice. NTZ also reduced inflammation in KO mice. Representative HE histologic appearance of oxyntic mucosa in the two KO groups compared to WT/PEG are presented in Figure 2.

### 2.4. NTZ Reduced NE Cell Hyperplasia, Whereas SPEM Was Unaffected in KO Mice 

**Figure 3:**
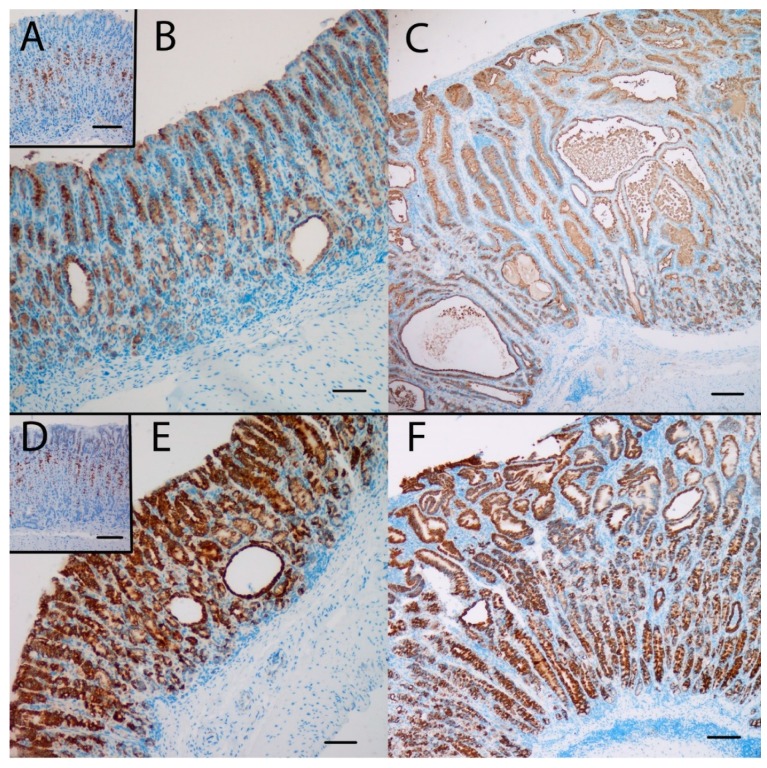
The SPEM marker clusterin was highly expressed at the protein (**A**,**B**,**C**) and the mRNA (**D**,**E**,**F**) level in the oxyntic mucosa of KO/PEG mice (**C** and **F**), compared to WT/PEG mice (**A** and **D**). Clusterin expression in KO mice was not affected by NTZ (**B** and **E**). Scare Bar = 100 µm.

**Figure 4:**
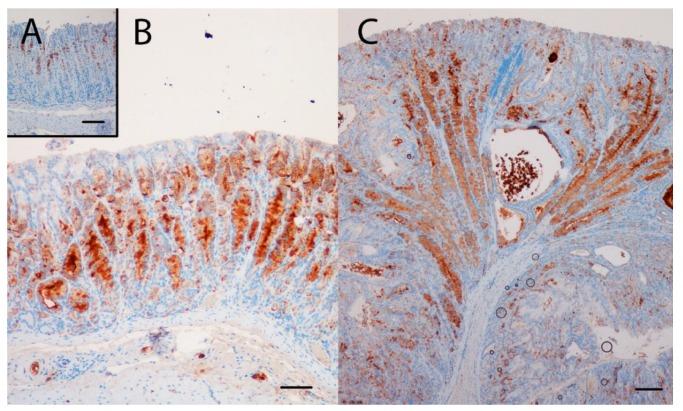
The SPEM marker TFF2 was highly expressed in the oxyntic mucosa of KO/PEG (**C**) and KO/NTZ mice compared to WT/PEG mice (**A**). TFF2 expression in KO mice was not affected by NTZ administration (**B**). Scare Bar = 100µm..

Gene expression analysis was performed to examine the relative expression of NE and SPEM related genes. This showed that numerous genes previously reported to be associated with SPEM were overexpressed in KO/PEG versus WT/PEG mice, including *tff2*, *clu*, *muc6*, *cd44* and *wfdc2*, with no significant change in KO mice receiving NTZ (Table 2). It was confirmed by both IHC and ISH that KO/PEG mice had pronounced expression of the SPEM markers TFF2 and clusterin in the gastric corpus [23] (Figure 3 and Figure 4), and that the SPEM did not seem to be affected by NTZ. There was also overexpression of markers of intestinalizing transcripts such as *cftr* and *muc4*. Only two (*muc13* and *muc5b*) of the SPEM associated genes overexpressed in KO/PEG mice compared to WT/PEG mice were reduced by NTZ. Expression of the mouse chief cell markers *pgc* (pepcinogen C), *gif* (intrinsic factor), *anpep* (Alanyl Aminopeptidase) and *Bhlha15* (MIST1) was substantially lower in KO/PEG than WT/PEG mice but was also not affected by NTZ with the exception of *anpep.*

**Figure 5:**
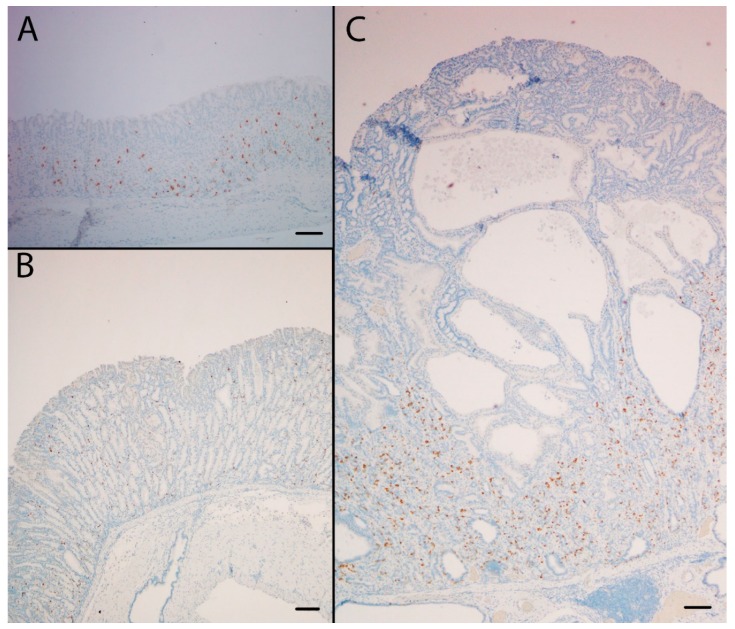
The NE marker CgA was highly expressed in the oxyntic mucosa of KO/PEG mice (**C**) compared to WT/PEG mice (**A**). Hyperplasia of NE cells in KO mice was reduced by NTZ in KO/NTZ mice (**B**). Scare Bar = 100 µm.

Conversely, NTZ significantly reduced the volume density of CgA-positive cells in KO mice (Figure 1F and Figure 5), demonstrating the effect of NTZ on NE cells. Gene expression analysis demonstrated that NTZ reduced expression of the general NE marker *chga* (CgA) as well as the ECL cell markers *hdc* (HDC), *slcl18a2* (VMAT-2) and *cckbr* (CCKBR) in KO mice, whereas expression of the D cell marker *sst* (somatostatin) was increased (Table 3). Expression of the general NE markers *eno2* (NSE) and *uchl1* (PGP9.5) and the enterochromaffin (EC) cell marker *tph1* (tryptophan hydroxylase) were, however, not affected by NTZ in KO mice.

### 2.5. Global Gene Expression Profiles Were Influenced More by Genotype Than Administration of NTZ 

**Figure 6:**
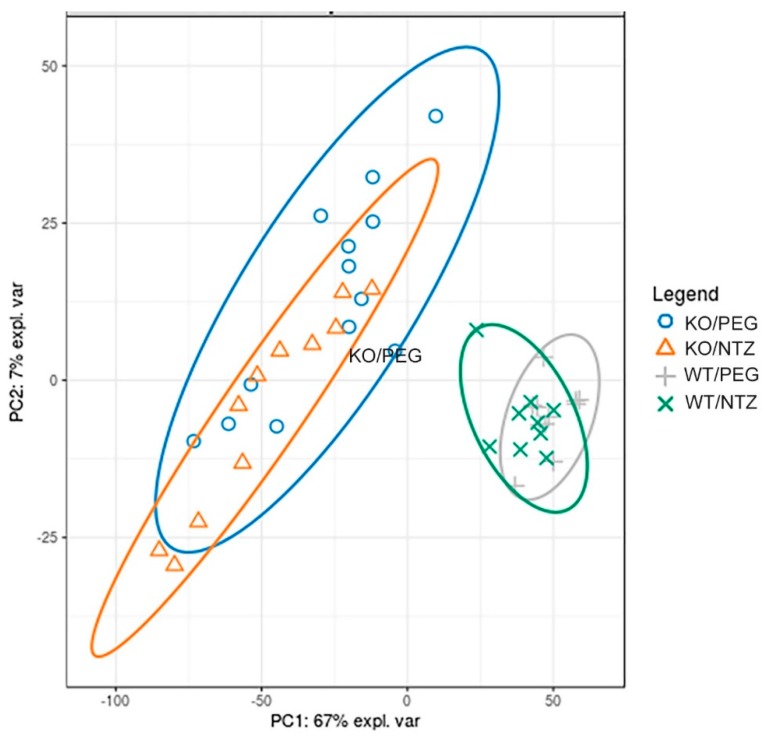
Global gene expression analysis of KO and WT mice given NTZ or PEG illustrated by at principal component analysis plot. Genotype affects global gene expression more than NTZ.

The global gene expression illustrated by a principal component analysis (Figure 6) displayed separation of both KO groups from the WT groups, with only minor separation between the NTZ and PEG groups. Thus, the global gene expression was influenced by genotype to a larger extent than by NTZ. The 20 most differentially expressed genes are shown in Table 4. Only three genes were differentially expressed in WT/PEG mice versus WT/NTZ mice (Supplementary Table S1).

## 3. Discussion

Various animal models with gastric hypoacidity and/or hypergastrinemia have been used to study carcinogenesis of the human gastric corpus and fundus [24]. Animal models have the potential to delineate disease mechanisms and numerous rodent models have also been used to study presumed premalignant changes of gastric corpus [16]. In the current study, we have examined the effects of the gastrin receptor antagonist NTZ on the oxyntic mucosa of anacidic and hypergastrinemic H^+^/K^+^ATPase beta subunit KO mice with particular focus on NE cell hyperplasia and SPEM. We found that NTZ reduced the pronounced hypertrophy of the oxyntic mucosa as well as intramucosal cysts and invasions below the muscularis mucosae. An antral gastrin receptor has been reported [25], but a trophic effect of hypergastrinemia or effects of gastrin receptor blockade with NTZ on antral mucosal thickness was not seen. NTZ reduced the volume density of NE cells as well as gene expression of several NE markers in the oxyntic mucosa of KO mice. Gene expression of the ECL cell markers HDC (gene *hdc*), VMAT-2 (gene *slcl18a2*) and CCKBR (gene *cckbr*) were reduced, as expected from studies localizing the CCKB-R on the ECL cell [26]. The expression of tryptophan hydroxylase (gene *Tph1*), which is expressed in EC cells, was not affected by NTZ in agreement with previous studies showing that EC cells are not stimulated by gastrin [27]. Interestingly, the expression of the D cell marker somatostatin (gene *sst*) was increased by NTZ in KO mice. This is in concordance with gastrin having a negative trophic effect on the D cells in the stomach [27,28] and may reflect simultaneous augmentation of a compensatory mechanism. Previously, we have found that NTZ prevents development of neoplasia in the gastric corpus in hypergastrinemic cotton rats [29]. Similarly, carcinogenesis in *M. natalensis* is enhanced by the histamine 2 receptor antagonist (H2RA) loxtidine [30], but inhibited by NTZ [31] and likewise gastric carcinogenesis in hypergastrinemic transgenic INS-GAS mice is inhibited by NTZ [32]. In patients with hypergastrinemia due to chronic atrophic gastritis, we have reported that NTZ can eradicate (type 1) ECL cell NE tumors (NETs) [33,34,35]. Interestingly, patients with chronic atrophic gastritis have an increased risk of both gastric adenocarcinomas and NETs [36,37,38]. Hypergastrinemia has also been found to be a risk factor for subsequent development of gastric cancer [8]. More recently, several epidemiological studies reported that patients using PPIs area at increased risk of gastric adenocarcinoma [5,6,7,39], whereas H2RA users did not. The studies combined therefore suggest that hypergastrinemia, a common factor in the mentioned animal models and human conditions with increased cancer risk, is pivotal in carcinogenesis of the gastric corpus and fundus [8,9].

Adenocarcinomas of the intestinal type after Lauréns classification [40] decline in Western populations [41] and the incidence is closely related to the prevalence of *Helicobacter pylori (H. pylori)* infection. IM is a proposed precursor lesion of intestinal type adenocarcinomas [42] and there is considerable interest for IM in risk stratification of patients undergoing gastroscopy, as well as in animal models to study mechanisms in the premalignant mucosa. More recently, SPEM has also been suggested to be a precursor lesion of gastric adenocarcinomas of intestinal type [43], which may be viewed as either an alternative or a supplementary hypothesis to the previously proposed Correa cascade [42]. In epidemiologic studies, antral *H. pylori* infection protects against gastric cancer [44] and it therefore seems paradoxical that *H. pylori*-induced IM in the antrum could be a precursor lesion of cancer. However, *H. pylori* infection of the oxyntic mucosa with atrophy, which leads to hypoacidity and pronounced hypergastrinemia, increases the risk of cancer considerably [45]. A corresponding risk consideration concerning isolated antral IM has gained acceptance and endoscopic surveillance is not recommended in the most recent clinical guidelines in Europe [46,47].

Within the described context, it was of particular interest to further examine the role of gastrin in SPEM development. We have previously found that the SPEM marker clusterin is highly expressed in ECL cells of normogastrinemic rats, but in rats with hypergastrinemia due to PPI administration, clusterin expression was considerably increased, through a dominant shift in the expression towards cells of the mucous neck-chief cell lineages. [23]. Moreover, clusterin was found to be highly upregulated in mucus neck and SPEM cells of the H^+^/K^+^ATPase beta subunit KO mice of different ages [23]. However, the finding of accelerated SPEM development in gastrin KO mice given DMP-777 [17] demonstrates that SPEM development in mice cannot depend entirely on gastrin. In the current study, KO/PEG mice had pronounced expression of SPEM in the gastric corpus and numerous genes previously reported to be associated with SPEM were differentially overexpressed compared to WT/PEG mice. The genes overexpressed included SPEM-defining *clu*, *tff2* and *muc6*, as well as *wfdc2* [48] and *cd44* [49] which also have been identified as markers of SPEM and do not occur in the normal gastric corpus. However, when comparing KO/PEG and KO/NTZ groups NTZ neither affected the existence of SPEM nor the expression of previously reported SPEM associated genes, as assessed both histologically and on protein and transcriptional level. 

We have previously described SPEM in oxyntic mucosa of 3-month-old H^+^/K^+^ATPase beta subunit KO mice [23]. Others have reported mice of age 35 days to be depleted of mature chief cells based on morphological criteria in HE stained sections [50]. The phenomenon was independent of hypergastrinemia since the same was found in mice double KO for gastrin and H^+^/K^+^ATPase beta subunit [50]. Loss of morphologically normal mature chief cells is one of the characteristics of SPEM, which might indicate that SPEM is present very early in oxyntic mucosa of this mouse model. Exactly when this occurs, and whether it is distinguishable from glands of the embryonic and juvenile stomach described by others [51,52], has not been investigated. In the current study, we found that the KO/PEG mice (at age 13 months) expressed only negligible amounts of several chief cell markers compared to WT/PEG mice, and the expression was unaffected by NTZ. Since KO mice depleted of mature chief cells still develop SPEM, mature chief cells may not be the origin of SPEM in the current mouse model. Expressions of Xbp1 and Mist1 (gene *Bhlha15*) were low in KO mice and these are important for chief cells to form zymogen-containing vesicles [53,54]. In the current mouse model, one could speculate that SPEM develops from the large proportion of cells previously described as immature cells in young KO mice [50], possibly represented chief cells devoid of granules. Alternatively, the observations could support the theory that SPEM is derived from isthmus stem cells [55]. 

The term SPEM is viewed by many as synonymous with the older terms “pyloric metaplasia”, “pseudopyloric metaplasia” or “antralization” and the actual alterations may be considered a common response to glandular injury observed in both mice and humans. Furthermore, ulcer-associated cell linage (UACL) has also been viewed as similar reparative linages and is observed in reparative processes along the gastrointestinal tract, including at the edges of gastric ulcers [56] and also in inflammatory bowel disease [57,58]. Also, one of the highly expressed markers of gastric SPEM, clusterin, seems to have subtle protective roles on oxyntic mucosa [59]. These observations combined suggest that SPEM should not be considered merely a preneoplastic lesion. Accordingly, it has been argued that SPEM could be an phenomenon associated with carcinogenesis, as a form of mucosal injury, not a precursor of cancer [60], and that SPEM and IM could be commensals for a neoplastic process rather than true direct precursors [43]. Reported findings from animal models of IM and SPEM have led to conflicting hypotheses of the cellular origin of the lesions, which may be either chief cells [61] or isthmus stem cells [55]. It is notable that the ability of gastric metaplasia to progress to invasive cancer seems to be lacking in the more than 20 mouse models utilized to study this, which also questions the validity of studies aiming to examine premalignant changes [16]. We propose that further studies of dysplasia and SPEM in patients with chronic atrophic gastritis treated long-term with NTZ [33,35] could provide more information about the roles of SPEM and hypergastrinemia in human carcinogenesis. 

In conclusion, the gastrin receptor antagonist NTZ prevented mucosal hypertrophy, intramucosal cysts, invasions of the muscularis mucosae as well as NE cell hyperplasia in the gastric corpus of H^+^/K^+^ATPase beta subunit KO mice. However, SPEM is found in oxyntic mucosa independently of gastrin signaling and the presence of SPEM seems unrelated to the presumed premalignant changes caused by hypergastrinemia in this animal model. The clinical implications of the current findings could be clarified through further studies of patients treated with NTZ.

## 4. Materials and Methods

### 4.1. Animals and Genotyping

H^+^/K^+^ATPase beta subunit KO mice, originally with a BalbC/black6 [22] background and later BalbC [62], were back-crossed 8 times onto BalbC mice (Møllegaard, Skensved, Denmark) before study start. DNA was isolated from tail samples using the high pure PCR template preparation kit, according to the manufacturer’s instruction (11796828001, Roche Diagnostics, Indianapolis, IN, USA). A PCR assay was set up to genotype mice and was used to distinguish between homozygous KO mice (-/-), heterozygous mice (+/-) and homozygous WT mice (+/+). For detection of H^+^/K^+^ATPase WT alleles, specific primers (forward primer: GGACCAACTGACTTCTGGGA and reverse primer: ACCTGCATGGCAGTCTCTCT, product length: 472 bp) were based on H^+^/K^+^ATPase beta subunit (*Atp4b*) sequence (Ensembl: ENSMUSG00000031449) and located approximately 100 bp upstream and downstream of exon 1 (the target for the homologous recombination). A PCR product with size of 472 bp would appear only if the WT allele was present. Inserted construct (PKG-neo) alleles were detected using specific primers selected from the PKG-neo sequence [22] (forward primer: AGACAATCGGCTGCTCTGAT and reverse primer: ATACTTTCTCGGCAGGAGCA, product length: 261 bp) and a PCR product with the size 261 bp would only appear if the KO allele was present. All variants were genotyped by combining these two PCR reactions using the FastStart PCR Master kit according to the manufacturer´s instruction (Roche Diagnostics), and visually inspected after gel electrophoresis separation and ethidium bromide staining.

For the experiments, we used female H^+^/K^+^ATPase KO mice (*n* = 21) and WT controls (*n* = 21) 1 month of age. They were housed in cages with aspen woodchip bedding (B&K Universal Ltd., Hull, UK). The room temperature was 24 ± 1 °C with a relative humidity of 40-50% and a 12h light/dark cycle. RM1 (E) diet (SDS, Essex, UK) and tap water were provided *ad libitum*. The study was approved by the Norwegian National Animal Research Authority.

### 4.2. Study Design

The drug netazepide (NTZ), previously named YF476 (Hammersmith Medicines Research, London, UK), has been shown to be a potent and highly selective competitive antagonist of the gastrin/CCKB receptor (CCKBR) [63]. It was dissolved to a concentration of 12 mg/mL in polyethylene glycol 300 (PEG) and given as subcutaneous injections at a dose of 40 mg/kg (80 µmol/kg) to *n* = 11 KO mice (KO/NTZ) and *n* = 10 WT mice (WT/NTZ) from age 1 month, once every two weeks for 12 months. Animals in the two control groups received an equivalent volume of PEG (KO/PEG, *n* = 10 mice and WT/PEG, *n* = 11 mice). The toxic dose of NTZ have been studied by Ferring and in 13-week studies, the no-observable-adverse-effect level was 100 mg/kg/day in rats and in dogs [64]. 

### 4.3. Intragastric pH and Plasma Gastrin at Termination 

The intragastric pH was measured by using a pediatric pH catheter with the stomach in situ. The lowest pH obtained after searching the entire corpus and antral mucosa was recorded for each animal. The animals were killed by exsanguination from the inferior cava while anesthetized with 5% isoflurane with O_2_ and N_2_ in a ratio of 3:2 as carrier gas with total gas flow of 1 L/min. Plasma was separated and kept at −20 °C until gastrin analysis by radioimmunoassay as previously described [65].

### 4.4. Histopathology, Immunohistochemistry (IHC) and in situ Hybridization (ISH) 

After measuring intragastric pH, the stomach was removed, rinsed in saline, weighed, opened along the greater curvature and inspected macroscopically. Tissue samples for histopathology were taken longitudinally from the rumen to the pylorus on the major curvature and fixed in 4% formaldehyde before processing and paraffin embedding. Sections (4 μm) were cut and stained with hematoxylin and eosin (HE) and examined microscopically. Dysplasia was graded as none, mild, moderate or severe, and inflammation was graded according to the Updated Sydney Classification [66]. To assess trophic changes, mucosal thickness was determined using an ocular grid. Measurements were performed in areas without macroscopic abnormalities and where gastric crypts were visible in their full length. Corpus and antral mucosal thickness were measured at five locations in each animal and the means recorded. The number of cystic dilations and invasions through the muscularis mucosa per mm horizontal mucosa was counted in areas where the gastric glands were cut longitudinally and visible in their whole length.

Tissue sections used for IHC were processed through standard pretreatment, including de-paraffinization, rehydration and quenching of endogenous peroxidase. Antigen retrieval was achieved by 15 min boiling in buffer (pH 6.0 for clusterin and TFF2, pH 9 for CgA). The following primary antibodies and conditions were used: anti-chromogranin A (CgA) (ImmunoStar 20080, dilution 1:2000, incubation for 1h at room temperature), goat anti-clusterin (C-18, sc-6419, Santa Cruz Biotechnology Inc., TX, USA; dilution 1:1300, incubation overnight at 4 °C) and rabbit anti-Trefoil Factor 2 (TFF2) (13681-1-AP, Proteintech Europe, dilution 1:900, incubation overnight at 4 °C). The clusterin immunoreaction was visualized using incubation with biotinylated anti-goat immunoglobulins (BA-5000, Vector Laboratories Inc., CA, USA) (1:200) for 30 min at room temperature, followed by the Vectastain Elite ABC HRP kit (PK-6100 Standard, Vector Laboratories Inc., CA). The TFF2 and CgA immunoreactions were visualized using the rabbit/mouse EnVision-HRP/DAB+ kit (K5007, DAKO, Glostrup, Denmark). All dilutions were done with Tris Buffered Saline (TBS) (50 mM, pH 7.4) with 0.025% Tween and 1% Bovine Serum Albumin (BSA). Omitting the primary antibody or incubation with matching isotype immunoglobulin were negative controls. The volume densities of CgA-immunoreactive cells were calculated in all mice using a point counting method as described previously [67].

ISH for clusterin mRNA was done using the RNAscope 2.0 HD Reagent Kit (Brown) for FFPE tissue (310035, Advanced Cell Diagnostics Inc, CA, USA) and a custom probe (427891, Mm-Clu) for clusterin, according to the manufacturer’s protocol. All IHC and ISH sections were counterstained with hematoxylin. Images were captured using Nikon E400 microscope, DS-Fil U2 camera and NIS-Elements BR imaging software (Nikon, Melville, NY, USA), and further processed using ImageJ. 

### 4.5. Whole-Genome Gene Expression Analysis

Biopsies from the ventral part of the gastric corpus mucosa were frozen in liquid N_2_ and then homogenized in lysis buffer using an Ultra-Turrax rotating knife homogenizer. The RNeasy Mini kit (Qiagen, Germantown, MD, USA) was used for total RNA extraction, according to the manufacturer’s instructions. RNA concentration was measured using Qubit^®^ RNA HS Assay Kit on a Qubit^®^ 3.0 Fluorometer (Thermo Fisher Scientific Inc., Waltham, MA, USA). Integrity was assessed using Agilent RNA 6000 Pico Kit on a 2100 Bioanalyzer instrument (Agilent Technologies, Santa Clara, CA, USA).

RNA sequencing libraries were prepared using TruSeq Stranded mRNA kit (Illumina, San Diego, CA, USA) according to the manufacturer’s instructions. In brief, 2 µg total RNA was used as starting material. First, mRNA was purified from the total RNA using poly-T oligo-attached magnetic beads, follows by random fragmentation using divalent cations at 94 °C for 4 min. First and second strand cDNAs were synthesized using random oligonucleotides and SuperScript II, followed by DNA polymerase I and RNase H. Exonuclease/polymerase was used to produce blunted overhangs. Illumina duel index adapter oligonucleotides were ligated to the cDNA after 3’ end adenylation. DNA fragments were enriched by 15 cycles of PCR reaction. The libraries were purified using the AMPure XP (Beckman Coulter, Inc., Indianapolis, IN, USA), quantitated by qPCR using KAPA Library Quantification Kit (Kapa Biosystems, Inc., Wilmington, MA, USA) and validated using Agilent High Sensitivity DNA Kit on a Bioanalyzer (Agilent Technologies, Santa Clara, CA, USA). The size range of the DNA fragments were measured to be in the range of app. 200–1000 bp and peaked around 285 bp.

Libraries were normalized and pooled to 2.0 pM and subjected to clustering on two NextSeq 500 high output flow cells. Finally, single read sequencing was performed for 75 cycles on a NextSeq 500 instrument (Illumina, Inc. San Diego, CA, USA), according to the manufacturer’s instructions.

Base calling was done on the NextSeq 500 instrument by RTA 2.4.6. FASTQ files were generated using bcl2fastq2 Conversion Software v2.17 (Illumina, Inc. San Diego, CA, USA). For each sample, kallisto (v0.42.4) was used to quantify GRCm38 transcripts and Sleuth was used for differential expression analysis [68]. Statistical significance was defined with a Wald test and Benjamini-Hochberg false discovery rate adjusted *P*-value < 0.05. The microarray experiments are Minimum Information About a Microarray Experiment (MIAME) compliant and have been deposited in the Gene Expression Omnibus (GEO) repository and assigned the GEO accession number GSE142513. The expressions of genes reported to be associated with SPEM [69,70] and NE cells [26,71,72,73,74] in previous publications were manually identified in the gene expression analysis.

## Figures and Tables

**Table 1:** Quantification of histopathological changes. Histopathological changes of the gastric corpus mucosa in KO mice given PEG (KO/PEG) or NTZ (KO/NTZ) and WT controls given PEG (WT/PEG) or NTZ (WT/NTZ).

	WT/PEG (*n* = 11)	WT/NTZ (*n* = 9)	KO/PEG (*n* = 9)	KO/NTZ (*n* = 11)	*p*-Value *
Intramucosal cysts (no/cm (median (range))	0.0 (0.0–0.0)	0.0 (0.0–0.0)	3.8 (1.6–6.8)	0.23 (0.0–1.3)	0.0001
Invasion of muscularis mucosa:					
No. of animals (n (%))	0	0	6 (66.7%)	6 (54.5%)	
No. of invasions/animal (median (range))	0	0	1 (0–4)	4 (0–12)	0.046
Inflammation score None Mild Moderate Severe	0 0 0 0	0 0 0 0	2 3 4 0	7 4 0 0	0.046

* *p*-value for comparisons between KO/PEG and KO/NTZ by Mann-Whitney-U test for intramucosal cysts and Fischer’s exact test for inflammation score. KO: H^+^/K^+^ATPase beta subunit knockout; PEG: polyethylene glycol; WT: wild-type; NTZ: netazepide; IQR: interquartile range.

**Table 2:** Expression of spasmolytic polypeptide-expressing metaplasia (SPEM)-related genes in the gastric corpus mucosa of KO/PEG versus WT/PEG mice and in KO/NTZ versus KO/PEG mice. Numerous of these genes were overexpressed in KO/PEG mice, but most of these genes were not affected by NTZ. Green: significant change (adjusted p-value (*q*-value) < 0.05); red: non-significant change (*q*-value > 0.05).

		KO/PEG vs. WT/PEG		KO/PEG vs. KO/NTZ	
Target_ID	Gene Symbol	log2 Fold Change	*q*-Value	log 2 Fold Change	*q*-Value
ENSMUST00000109344	*Wfdc2*	5.05	3.0814 × 10^−15^	−0.59	0.7467
ENSMUST00000115119	*Muc4*	5.03	4.0540 × 10^−33^	−0.26	0.4441
ENSMUST00000027675	*Pigr*	4.97	2.2607 × 10^−54^	0.04	0.9695
ENSMUST00000060833	*Gpa33*	4.18	4.7773 × 10^−11^	−0.01	0.9952
ENSMUST00000045706	*Cftr*	3.96	4.6493 × 10^−24^	−0.38	0.6774
ENSMUST00000017867	*Wfdc2*	3.84	8.4954 × 10^−51^	−0.49	0.3777
ENSMUST00000189314	*Muc6*	3.17	1.5584 × 10^−20^	−0.86	0.1857
ENSMUST00000033414	*Slc6a14*	3.10	7.2586 × 10^−9^	0.40	0.7606
ENSMUST00000023520	*Muc13*	3.03	3.6130 × 10^−19^	1.27	0.0156
ENSMUST00000166860	*Gpa33*	2.86	1.5576 × 10^−10^	0.34	0.7540
ENSMUST00000098668	*Ceacam1*	2.82	7.8390 × 10^−16^	−0.20	0.8293
ENSMUST00000165147	*Muc5b*	2.56	8.9582 × 10^−13^	1.35	0.0128
ENSMUST00000171080	*Fignl1*	2.49	0.0004	1.03	0.4641
ENSMUST00000189068	*Ly6a*	2.39	0.0001	−0.17	0.9288
ENSMUST00000022616	*Clu*	2.27	2.4223 × 10^−25^	−0.08	0.8968
ENSMUST00000061829	*Cd14*	2.20	2.2346 × 10^−50^	0.33	0.2595
ENSMUST00000024826	*Tff2*	2.05	5.3435 × 10^−5^	−0.63	0.2658
ENSMUST00000139156	*Akr1b10*	1.94	0.0068	0.16	0.9409
ENSMUST00000027366	*Vil1*	1.88	1.5339 × 10^−30^	0.17	0.6503
ENSMUST00000193391	*Il18r1*	1.79	0.0001	−0.16	0.9019
ENSMUST00000066723	*Lgals4*	1.67	1.6543 × 10^−23^	0.38	0.2317
ENSMUST00000005218	*Cd44*	1.67	0.0313	1.23	0.3969
ENSMUST00000011178	*Slc5a1*	1.59	2.2000 × 10^−14^	−0.24	0.5989
ENSMUST00000056117	*Itga2*	1.44	0.0005	0.82	0.2941
ENSMUST00000098666	*Ceacam1*	1.41	0.0017	0.68	0.4358
ENSMUST00000076648	*Fcgbp*	1.30	0.0050	0.67	0.4623
ENSMUST00000027015	*Casp1*	1.29	3.1977 × 10^−5^	0.43	0.4968
ENSMUST00000062451	*Muc6*	1.23	0.0004	−0.67	0.3267
ENSMUST00000017743	*Krt20*	1.18	2.5460 × 10^−7^	0.28	0.5581
ENSMUST00000161870	*Glipr1*	1.17	0.0058	0.01	0.9985
ENSMUST00000025647	*Pga5*	1.08	0.0007	0.12	0.8895
ENSMUST00000148005	*Mmp12*	0.87	0.0238	0.38	0.6441
ENSMUST00000032800	*Tyrobp*	0.87	1.0831 × 10^−7^	0.21	0.5450
ENSMUST00000200880	*Cxcl17*	0.73	1.0409 × 10^−13^	0.01	0.9697
ENSMUST00000111194	*Cd44*	0.59	0.0029	−0.03	0.9541
ENSMUST00000038069	*Ceacam10*	0.53	0.0009	0.28	0.3423
ENSMUST00000017530	*Traf4*	0.40	5.1315 × 10^−6^	−0.03	0.8775
ENSMUST00000034304	*Hsd17b2*	0.33	0.04474	−0.48	0.3913
ENSMUST00000049004	*Anpep*	−0.42	0.0000	0.01	1.7276
ENSMUST00000060747	*Bhlha15*	−1.19	2.0159 × 10^−11^	0.26	0.4639
ENSMUST00000025585	*Gif*	−2.11	1.3159 × 10^−6^	−0.48	0.6294
ENSMUST00000149623	*Xbp1*	−2.24	2.2991 × 10^−7^	0.36	0.7162
ENSMUST00000144955	*Pgc*	−10.43	6.6393 × 10^−80^	−0.86	0.2757

**Table 3:** Expression of NE markers in gastric corpus mucosa of WT/PEG versus KO/PEG mice and KO/ NTZ versus KO/PEG mice. General NE and ECL cell markers were overexpressed in KO mice and downregulated by NTZ. Green: significant change (adjusted p-value (*q*-value) < 0.05); red: non-significant change (*q*-value > 0.05).

Target_ID	Gene Symbol	KO/PEG vs. WT/PEG		KO/NTZ vs. KO/PEG	
		Log2 Fold Change	*q*-Value	Log 2 Fold Change	*q*-Value
ENSMUST00000021610	*Chga*	1.02	8.3251 × 10^−21^	−0.81	4.4936 × 10^−11^
ENSMUST00000028838	*Hdc*	0.91	4.3816	−1.35	2.1732 × 10^−16^
ENSMUST00000026084	*Slc18a2*	0.35	0.0376	−0.58	0.0179
ENSMUST00000033189	*Cckbr*	1.02	1.8375 × 10^−10^	−0.70	0.0016
ENSMUST00000004480	*Sst*	−0.99	1.8398 × 10^−6^	0.78	0.0119
ENSMUST00000112476	*Eno2*	1.62	0.0109	−1.92	0.1330
ENSMUST00000031131	*Uchl1*	−1.02	0.0469	0.05	0.9709
ENSMUST00000107669	*Tph1*	2.16	6.5746 × 10^−8^	0.16	0.8868

**Table 4:** Global gene expression analysis in KO/PEG versus KO/NTZ and KO/PEG versus WT/PEG mice illustrating the effect of NTZ in KO mice. Differentially expressed genes in the gastric corpus mucosa of KO/NTZ mice in comparison with KO/PEG mice sorted by ascending adjusted *p*-value (*q*-value). Green: significant change (adjusted *p*-value (*q*-value) < 0.05); red: non-significant change (*q*-value > 0.05).

Target_ID	Gene Symbol	KO/PEG vs. KO/NTZ		WT/PEG vs. KO/PEG	
		Log2 Fold Change	*q*-Value	Log2 Fold Change	*q*-Value
ENSMUST00000049209	*Gc*	−1.90	1.5098 × 10^−21^	0.42	0.0391
ENSMUST00000028838	*Hdc*	−1.35	2.1707 × 10^−16^	0.91	4.3816 × 10^−9^
ENSMUST00000028826	*Chgb*	−1.05	2.8712 × 10^−14^	0.68	1.6566 × 10^−7^
ENSMUST00000021610	*Chga*	−0.81	4.4903 × 10^−11^	1.02	8.3251 × 10^−21^
ENSMUST00000159861	*Pappa2*	−1.41	2.5024 × 10^−8^	0.35	0.1301
ENSMUST00000017488	*Vtn*	−0.96	1.0605 × 10^−21^	2.15	0.0000
ENSMUST00000125379	*Ndufa12*	−3.83	1.2607 × 10^−7^	−1.79	0.0052
ENSMUST00000128285	*Tfrc*	−0.63	3.5205 × 10^−7^	0.87	1.6624 × 10^−17^
ENSMUST00000023583	*Ahsg*	−1.84	5.4799 × 10^−7^	0.72	0.0273
ENSMUST00000050397	*Sprr2f*	−2.15	6.5281 × 10^−7^	1.27	0.0006
ENSMUST00000019063	*Tm4sf5*	1.00	1.5501 × 10^−6^	−0.32	0.0939
ENSMUST00000114474	*Plet1*	0.61	1.5973 × 10^−6^	0.85	3.9671 × 10^−16^
ENSMUST00000041096	*Pcsk1n*	−1.16	2.2102 × 10^−6^	−0.19	0.3898
ENSMUST00000022075	*Pcsk1*	1.14	2.5357 × 10^−6^	0.06	0.7982
ENSMUST00000039559	*Thbs1*	1.12	2.7234 × 10^−6^	0.52	0.0115
ENSMUST00000021813	*Barx1*	−0.56	8.4929 × 10^−6^	−0.04	0.7504
ENSMUST00000063278	*Agt*	0.68	1.0863 × 10^−5^	1.62	1.0802 × 10^−40^
ENSMUST00000041416	*Vnn1*	1.12	1.8311 × 10^−5^	3.09	3.2814 × 10^−52^
ENSMUST00000108858	*Sparc*	0.47	2.5356 × 10^−5^	0.11	0.2421
ENSMUST00000025356	*Mal2*	0.52	3.0993 × 10^−5^	0.04	0.7033

## Supplementary Materials

Supplementary materials can be found at https://www.mdpi.com/1422-0067/21/3/927/s1.

## Abbreviations

CgA	Chromogranin A
EC	Enterochromaffin
ECL	Enterochromaffin-like
GEO	Gene Expression Omnibus
GRP	Gastrin-releasing peptide
GRP-R	Gastrin-releasing peptide receptor
HDC	Histidine decarboxylase
IHC	Immunohistochemistry
IM	Intestinal metaplasia
ISH	In-situ hybridization
KO	Knockout
MIAME	Minimum Information About a Microarray Experiment
NE	Neuroendocrine
NET	Neuroendocrine tumour
NTZ	Netazepide
PEG	Polyethylene glycol
PPI	Proton pump inhibitor
SPEM	Spasmolytic polypeptide-expression metaplasia
WT	Wild-type
