# Supplementary material for: Gastric Corpus Mucosal Hyperplasia and Neuroendocrine Cell Hyperplasia, but not Spasmolytic Polypeptide-Expressing Metaplasia, Is Prevented by a Gastrin Receptor Antagonist in H+/K+ATPase Beta Subunit Knockout Mice
Source: Int J Mol Sci. 2020 Jan 31;21(3):927. doi: 10.3390/ijms21030927 (PMC7037105; doi:10.3390/ijms21030927)
Supplement: Supplementary file 1 [file ijms-21-00927-s001.pdf]

Supplementary Table S1. Global gene expression analysis in WT/PEG vs WT/NTZ mice. Only three genes were differentially expressed with adjusted p-value (q-value) < 0.05 in the gastric corpus mucosa. A negative Log2 fold change signifies a significantly lower expression in the WT/NTZ vs WT/PEG group. Green: significant change (qval > 0.05).

Target Id	***Gene symbol***	**Log2 fold change**	**p-value**
ENSMUST00000109775	Apol9b	−2.09	0.0143
ENSMUST00000129733	Tpm1	−3.10	0.0143
ENSMUST00000033738	Trex2	−1.43	0.0143
